# Exploring the Link Between Biotin Metabolism and *Brucella* Virulence: A Study on *BioA*

**DOI:** 10.3390/pathogens15050473

**Published:** 2026-04-27

**Authors:** Donghui Liu, Heng Quan, Mengyao Liu, Lingling Xiao, Lei Jiao, Xiaowei Gong, Qiaoying Zeng, Qiwei Chen

**Affiliations:** 1 College of Veterinary Medicine, Gansu Agricultural University, Lanzhou 730070, China; donghuiliu63@163.com; 2 State Key Laboratory of Animal Disease Control and Prevention, Lanzhou Veterinary Research Institute, Chinese Academy of Agricultural Sciences, College of Animal Medicine and Biosafety, Lanzhou University, Lanzhou 730030, China; quanheng0517@163.com (H.Q.); lmy11292025@163.com (M.L.); anion0418@163.com (L.X.); gongxiaowei@caas.cn (X.G.); 3 The Third Research Department, Lanzhou Institute of Biological Products Co., Ltd., Gansu Provincial Vaccine Technology Innovation Center, Lanzhou 730046, China; jiaolei_1984@163.com

**Keywords:** *Brucella abortus*, *BioA*, biotin metabolism, virulence, type IV secretion system

## Abstract

Background: The intracellular pathogen *Brucella* requires biotin for survival, yet the role of its de novo synthesis intermediate enzyme, *BioA*, in virulence remains undefined. This study investigates the contribution of *BioA* to the pathogenicity of *Brucella abortus*. Methods: We constructed a *ΔBioA* mutant in *Brucella abortus* 104M via homologous recombination and characterized its phenotype using growth assays, electron microscopy, macrophage infection models, and murine splenic colonization. Virulence gene expression was quantified by RT-qPCR. Results: The *ΔBioA* mutant exhibited severe growth auxotrophy in a biotin-deficient medium and displayed damaged outer membrane integrity. Furthermore, intracellular survival in macrophages was reduced by approximately 95% compared to the wild-type strain at 48 h post-infection. Notably, mice infected with the mutant showed a significant decrease in both splenic bacterial loads and spleen weight at 3 weeks, concomitant with a marked downregulation of VirB type IV secretion system (T4SS) genes. Conclusions: This study is the first to identify *BioA* as a critical nexus linking biotin metabolism to *Brucella* virulence. We demonstrate that *BioA* deficiency attenuates pathogenicity by impairing both structural integrity and the transcription of key virulence-related genes (VirB operon), thereby nominating *BioA* as a novel and promising target for anti-brucellosis interventions.

## 1. Introduction

Biotin, namely vitamin H or vitamin B7, also known as coenzyme R, with the chemical name 5-[(3aS,4S,6aR)-2-oxohexahydro-1H-thieno[3,4-d]imidazol-4-yl]pentanoic acid, is necessary for the growth and pathogenicity of many pathogenic bacteria [1]. Biotin is a coenzyme of carboxylase, which is involved in the carboxylation, decarboxylation and transcarboxylation of fatty acid biosynthesis, gluconeogenesis and amino acid metabolism [2]. All organisms need biotin, but mammals cannot synthesize it by themselves [3]. They can only obtain biotin through their diet and intestinal microbes. In contrast, prokaryotes can synthesize biotin independently. This difference renders the bacterial biotin synthetic pathway a promising target for the development of antibacterial agents.

In most prokaryotes, genes encoding biotin synthetic enzymes are typically organized into operons [4]. The de novo synthesis of biotin is mainly divided into two major steps, including the formation of precursor pimelate monoacyl CoA and the conversion of biotin through pimelate monoacyl CoA. The early stage of the biotin synthesis pathway is differentiated into different bacteria, and can be roughly divided into the classical BioC- BioH pathway (Escherichia coli) [5], the BioI–BioW pathway (*Bacillus subtilis*) [6] and the non-classical BioZ pathway (Alphaproteobacteria) [7]. Recent biochemical and structural studies on the BioZ pathway have greatly advanced the understanding of the diversity of biotin synthesis mechanisms [8]. The late stage of the biotin synthesis pathway is relatively conserved. Notably, 7-keto-8-aminononanoic acid (KAPA) is the first intermediate product of this process, which is assembled from L-alanine to form pimelate monoacyl CoA by 7-keto-8-aminononanoic acid synthase (BioF). The 7,8-diaminononanoic acid synthetase (BioA) can transfer the amino group of S-adenosyl-L-methionine (SAM) to KAPA, thus generating 7,8-diaminononanoic acid (DAPA). Desthiobiotin synthase (BioD) catalyzes the closure of the urea ring to form desthiobiotin (DTB), and biotin synthase (BioB) catalyzes the closure of thiophene heterocycle to form biotin. De novo synthesis of biotin is a biological process that consumes a lot of ATP. Therefore, some bacteria have evolved a set of auxiliary pathways for biotin uptake from the environment, that is, BioY-mediated transport pathways [9,10]. Due to the high energetic cost of de novo biotin synthesis, bacteria precisely control the transcription of biotin synthetase through regulatory factors such as BirA [11], BioR [12], and BioQ [13]. This strict regulatory mechanism highlights the central role of biotin metabolism in the physiological activities of bacteria.

*Brucella* is a *α-proteobacteria* and a facultative intracellular parasitic bacterium that can survive within host cells by evading host immune defenses, causing brucellosis and seriously endangering human health and the development of animal husbandry [14]. Intracellular survival is a critical virulence determinant for *Brucella* [15]. Recent studies have found that the host restricts the accessibility of essential micronutrients (such as zinc, manganese, iron and copper) through a nutritional immunity strategy to curb the proliferation of intracellular pathogens [16]. In this evolutionary game with the host, *Brucella* has evolved sophisticated metabolic adaptation mechanisms. Among these, biotin, as an essential cofactor of carboxylase, participates in core pathways such as fatty acid synthesis, gluconeogenesis, and branched-chain amino acid metabolism, and becomes a key metabolic hub for maintaining intracellular homeostasis. Therefore, exploring the correlation between biotin metabolism and the pathogenicity of *Brucella* has important scientific significance. Since mammals do not possess similar biotin-metabolism-regulatory mechanisms as pathogenic bacteria, and bacteria within macrophages are subjected to hypoxia and nutrient deprivation stress, studying the functions of genes related to *Brucella*’s de novo synthesis or transport of biotin can provide candidate targets for inhibiting its intracellular survival and developing anti-*Brucella* drugs. At present, the research on the genes related to biotin metabolism in *Brucella* is still in its infancy. The only reported study found that the regulatory factor BioR in *Brucella* is located upstream of the biotin transporter gene *BioY* and the biosynthetic enzyme genes *BioB*/*F*/*D*/*A*/*Z* operon, and it has inhibitory effects on both the de novo synthesis and transport of biotin [12]. However, this study did not conduct knockout verification of the biotin-synthesis-related genes in *Brucella*, and the precise regulatory network and physiological functions of the bacteria need to be further elucidated.

In the conserved late pathway of biotin synthesis, the pyridoxal 5′-phosphate (PLP)-dependent aminotransferase encoded by the *BioA* gene catalyzes a pivotal transamination reaction converting KAPA to DAPA. This step is indispensable for the formation of the biotin precursor. Since host cells lack the complete enzymatic machinery for de novo biotin synthesis, particularly the enzyme required for this specific transamination, *BioA*-deficient strains cannot compensate for this metabolic block by utilizing host-derived precursors. This makes *BioA* an ideal target for studying the relationship between metabolic dependence and bacterial virulence. In *Mycobacterium tuberculosis*, deletion of *BioA* impairs bacterial survival and abrogates mouse infection [17]. Moreover, various small-molecule compounds have been reported to exhibit anti-tuberculosis activity by targeting *BioA* [18,19]. For instance, through structure-based virtual screening, researchers have successfully identified a novel small-molecule inhibitor, A65, that can effectively target the active site of *BioA* in *M. tuberculosis* [19]. The *BioA* inhibitor C48 reduced the burden of *M. tuberculosis* in the lungs and spleen of a mouse model, validating in vivo for the first time a proof of concept for biotin biosynthesis as a *M. tuberculosis* treatment strategy [20]. This inhibitor can inhibit the activity of the *BioA* enzyme and the growth of the pathogen, and possesses characteristics similar to those of drugs. These findings provide a solid theoretical basis for conducting similar research in *Brucella*. Accordingly, this study aims to systematically investigate the effects of the biosynthesis gene *BioA* in the later stage of biotin synthesis on the physiological functions, intracellular survival, and pathogenicity of *Brucella*, with the expectation of seeking new breakthroughs in the development of new preventive vaccines and therapeutic drugs for brucellosis.

## 2. Materials and Methods

### 2.1. Reagents

Tryptic soy agar (TSA), tryptic soy broth (TSB), and Luria–Bertani medium (LB) were acquired from Difco Laboratories (Detroit, MI, USA). Dulbecco’s modified Eagle’s medium (DMEM) and fetal bovine serum (FBS) were supplied by Gibco Life Technologies (Rockville, MD, USA). TritonX-100, sucrose, gentamicin, and kanamycin were bought from Solarbio (Beijing, China). Streptavidin magnetic beads were provided by Beaver Beads (Suzhou, China). Avidin was purchased from Sangon Biotech (Shanghai, China). The RNA extraction kit and reverse transcription kit were bought from Promega Corporation (Madison, WI, USA).

### 2.2. Bacterial Strain, Plasmids, Primers, Cell, and Mice

*Brucella abortus* 104M (*B. abortus* 104M) was provided by the Third Research Department, Lanzhou Institute of Biological Products Co., Ltd., (Lanzhou, China). It was cultured on TSA or in TSB at 37 °C. To observe the growth of bacterial in a biotin-free environment, we used 1% streptavidin magnetic beads and avidin (2 μg/mL) to remove biotin from the medium. To validate the establishment of a biotin-free environment, the biotin-depleted medium was functionally verified using an *E. coli MG1655 ∆BioH* strain deficient in biotin biosynthesis genes [21]. This biotin-auxotrophic strain failed to grow in the treated medium, confirming that biotin was removed to a concentration below the minimal level required for growth. For selection or maintenance of the resistant strains, kanamycin (25 μg/mL) or 12% sucrose was added into the media. *Escherichia coli DH5α* (TransGen Biotech, Beijing, China) was used for cloning. For selection or maintenance of the resistant strains, kanamycin (50 μg/mL) was added into LB. The primers used in this study are shown in Supplementary Table S1. The murine macrophage cell lineage (RAW 264.7, obtained from Cell Resource Center, IBMS, CAMS/PUMC, Beijing, China) was propagated at 37 °C in DMEM lacking antibiotics, but augmented with 10% FBS and a 5% CO_2_ atmosphere. To remove *B. abortus* 104M outside the cells, gentamicin (25 μg/mL or 50 μg/mL) was added to the medium. Female specific-pathogen-free (SPF) BALB/c mice aged 5–6 weeks were bought from the Experimental Animal Center of Lanzhou Veterinary Research Institute (Lanzhou, China).

### 2.3. Construction of Gene Deletion and Complementation Strains

To delete the *BioA* gene cluster in the genome of the *B. abortus* 104M strain, the suicide plasmid PUC19-Kana-SacB-BioA was constructed via a one-step cloning strategy. First, upstream and downstream flanking fragments of *BioA* were amplified using *B. abortus* 104M genomic DNA as template. Then, the two fragments were assembled by overlapping extension PCR to generate one homologous recombination fragment. The purified PCR product was cloned into Hind III-digested PUC19-Kana -SacB using a clonexpress II one step cloning kit (Vazyme, Nanjing, China). The resulting suicide plasmids were electroporated into competent *B. abortus* 104M. The first homologous recombination was selected on TSA containing kanamycin, and the second recombination was selected on TSA containing 12% sucrose. Deletion strains were verified by PCR and sequencing. The *BioA* target gene fragment was amplified and cloned into the shuttle plasmid pBBR1MCS-2 using a one-step cloning strategy. To distinguish the complementation plasmid from the suicide plasmid (PUC19-Kana-SacB-BioA) used for gene deletion, the pBBR1MCS-2 vector was modified to encode an ampicillin resistance marker. The recombinant plasmid was electroporated into the deletion mutant. Transformants were selected on 25 μg/mL ampicillin-containing agar plates. Antibiotic pressure (25 μg/mL ampicillin) was maintained throughout all subsequent in vitro culture and assays to ensure plasmid retention. Genetically stable complemented strains were confirmed by PCR and sequencing.

### 2.4. Growth Analysis

The *B. abortus* 104M, the *ΔBioA* deletion mutant, and the *ΔBioA* complemented strain were cultured on TSA plates at 37 °C. A single colony of each strain was inoculated into 200 mL biotin-deficient TSB and a standard TSB medium, respectively. During incubation at 37 °C for 72 h, the optical density at 600 nm (OD_600_) was measured every 6 h using a spectrophotometer (Eppendorf, Hamburg, Germany). In vitro growth curves were generated based on the OD_600_ values. Each growth curve assay was performed with 3 independent biological replicates, and each replicate was measured in triplicate.

### 2.5. Electron Microscopy Analysis

To assess the potential effects of gene deletion on bacterial ultrastructure, the *B. abortus* 104M, *ΔBioA* and *cΔBioA* were subjected to scanning electron microscopy (SEM) and transmission electron microscopy (TEM) analysis. Briefly, bacterial cells from mid-logarithmic phase cultures were harvested by centrifugation and primarily fixed with 2.5% glutaraldehyde in 0.1 M PBS (pH 7.4) at 4 °C. The fixed cell pellets were washed with PBS and subsequently entrusted to Servicebio Biotechnology Co., Ltd., Wuhan, China, for all further specialized processing. Final imaging and data acquisition were performed using the company’s field-emission SEM and TEM instruments.

### 2.6. Acetyl-CoA and Malonyl-CoA Content Determination

The *B. abortus* 104M strain and *ΔBioA* mutant strain were cultured to the logarithmic growth phase, washed with PBS, and harvested. Bacterial quantification was performed by plate counting, and 1 × 10^9^ CFU bacteria were precisely harvested for subsequent experiments. After ultrasonic disruption of the bacterial cells, the supernatant was collected for subsequent testing. Acetyl-CoA content was determined using the Acetyl-CoA Content Assay Kit (Solarbio Science & Technology Co., Ltd., Beijing, China) according to the manufacturer’s protocol. Malonyl-CoA content was detected using the Microbial Malonyl-CoA Kit (Jiangsu Meimian Biotechnology Co., Ltd., Yancheng, China). Both assays were normalized based on the bacterial quantity (1 × 10^9^ CFU bacteria per sample) to eliminate variations in sample loading. All metabolite measurements were performed with 3 biological replicates, each of which included 3 technical replicates.

### 2.7. qRT-PCR

In order to detect the expression levels of different genes related to biotin synthesis, *B. abortus* 104M strains were cultured in normal TSB and biotin-free TSB to OD_600_ 0.6. Cells were collected and total RNA was extracted using an RNA prep Pure Cell/Bacteria Kit. Reverse transcription was performed using the RT Reagent Kit, and RT–PCR was performed using ChamQ SYBR qPCR Master Mix (Vazyme, Nanjing, China) on an ABI 7500 system. Data analysis used the 2^−ΔΔCt^ method. The relative expression levels of virulence genes in the *ΔBioA* strain and the *cΔBioA* strain were also determined using the same method. All primers for qRT-PCR are listed in Supplementary Table S1. Notably, 16S rRNA was used as the reference gene for qRT-PCR normalization to correct for variation in RNA extraction and reverse transcription efficiency. All qRT-PCR experiments were performed with 3 independent biological replicates, and each replicate was run in triplicate.

### 2.8. Macrophage Infection

Murine macrophage RAW 264.7 cells were seeded in 24-well plates (2 × 10^5^ cells per well) and infected with *B. abortus* 104M, the *ΔBioA* strain and the *cΔBioA* strain for 4 h at a multiplicity of infection of 100:1. Cells were incubated for 1 h with a cell culture medium containing 50 µg/mL gentamicin to eliminate extracellular bacteria, and then in a medium containing gentamicin (25 µg/mL) to avert continuous infection. RAW 264.7 cells were lysed in 0.5 mL of PBS-0.5% Triton X-100, and the lysates were plated on TSA to determine CFUs. Each macrophage infection assay was performed with 3 independent biological replicates, and each replicate contained 3 technical replicates (wells) per bacterial strain.

### 2.9. Residual Virulence Assay

The residual virulence of *Brucella* strains was evaluated by determining the median lethal dose (LD_50_) in a mouse model. Fresh cultures of *B. abortus* 104M were diluted in PBS to obtain five bacterial suspensions with concentrations of 1.5 × 10^9^ CFU/mL, 3.0 × 10^9^ CFU/mL, 6.0 × 10^9^ CFU/mL, 1.2 × 10^10^ CFU/mL, and 2.4 × 10^10^ CFU/mL. Similarly, fresh cultures of *ΔBioA* mutant strain were diluted to six concentrations: 3.0 × 10^9^ CFU/mL, 6.0 × 10^9^ CFU/mL, 1.2 × 10^10^ CFU/mL, 2.4 × 10^10^ CFU/mL, 4.8 × 10^10^ CFU/mL, and 9.6 × 10^10^ CFU/mL. Groups of 5 BALB/c mice (*n* = 5) each were inoculated intraperitoneally with 0.5 mL of one of the bacterial suspensions. All mice were observed for 7 days post-inoculation, and the LD_50_ was calculated based on mortality data.

### 2.10. Mouse Infection

Female BALB/c mice aged 5–6 weeks were subcutaneously inoculated with 5 × 10^7^ CFU of *B. abortus* 104M or the *ΔBioA* mutant [22]. At 1, 2, 3, and 4 weeks post-immunization, the mice’s body weights were measured, and their spleens were removed. Spleen weights were measured to evaluate splenomegaly, and the bacterial loads in the spleens were determined by homogenizing the tissues, serially diluting the samples, and plating them onto TSA media for colony-forming unit (CFU) enumeration. The mouse infection experiment was performed with 3 independent biological replicates, with 6 mice (*n* = 6) per bacterial strain per time point in each replicate.

### 2.11. Statistical Analysis

Experiments were performed for at least three independent biological replicates. The SPSS 22 software was used for statistical comparison. Results are presented as means ± standard deviation. Further analyses were performed using unpaired two-tailed *t*-tests, with Welch’s correction and one-way analysis of variation followed by Tukey’s multiple-comparison test. Welch’s *t*-test was specifically used for datasets with unequal variances. Probability (*p*) values < 0.05 were considered statistically significant.

## 3. Results

### 3.1. Biotin-Free Environment Can Affect the Expression of Genes Related to Biotin Synthesis in Brucella

**Figure 1:**
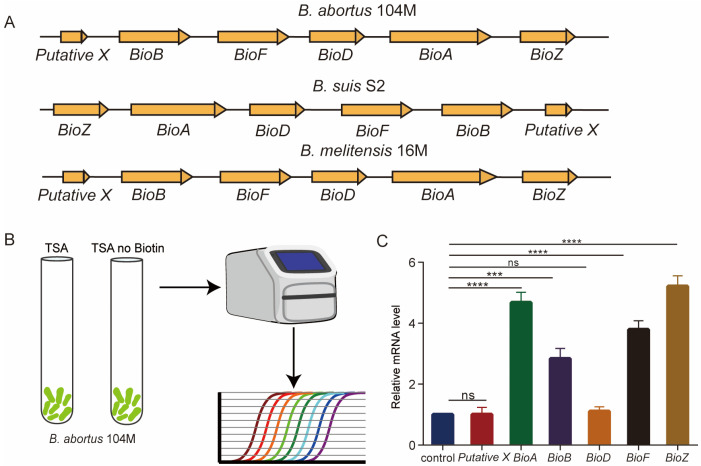
A biotin-free environment can affect the expression of genes related to biotin synthesis in *Brucella*. (**A**) The operon model of the genes related to biotin synthesis of *B. abortus* 104M, *B. suis S2* and *B. melitensis* 16M. (**B**) Detection model diagram of gene expression levels related to biotin synthesis in *B. abortus* 104M under biotin-deficient conditions. (**C**) Expression levels of biotin-synthesis-related genes in *B. abortus* 104M under biotin-deficient conditions. Data represent mean and standard deviation. *** *p* < 0.001, **** *p* < 0.0001 by one-way ANOVA followed by Dunnett’s multiple comparisons test.

The genes involved in biotin synthesis are located in a single operon, including *Putative X*, *BioF*, *BioA*, *BioD*, *BioB* and *BioZ* (Figure 1A). To examine the effect of biotin on *Brucella*, we designed qPCR primers for the late genes of *Brucella* biotin synthesis (Supplementary Table S1). The expression levels of late biotin synthesis genes of *Brucella* cultured in a normal TSB medium and a biotin-free TSB medium were detected (Figure 1B). Compared with *B. abortus* 104M grown in a normal TSB medium, the expression levels of *BioA*, *BioB*, *BioF* and *BioZ* in *Brucella* grown in a biotin-free TSB medium was significantly upregulated (Figure 1C).

### 3.2. Construction of ∆BioA Mutant and Complementary Strain in B. abortus 104M

**Figure 2:**
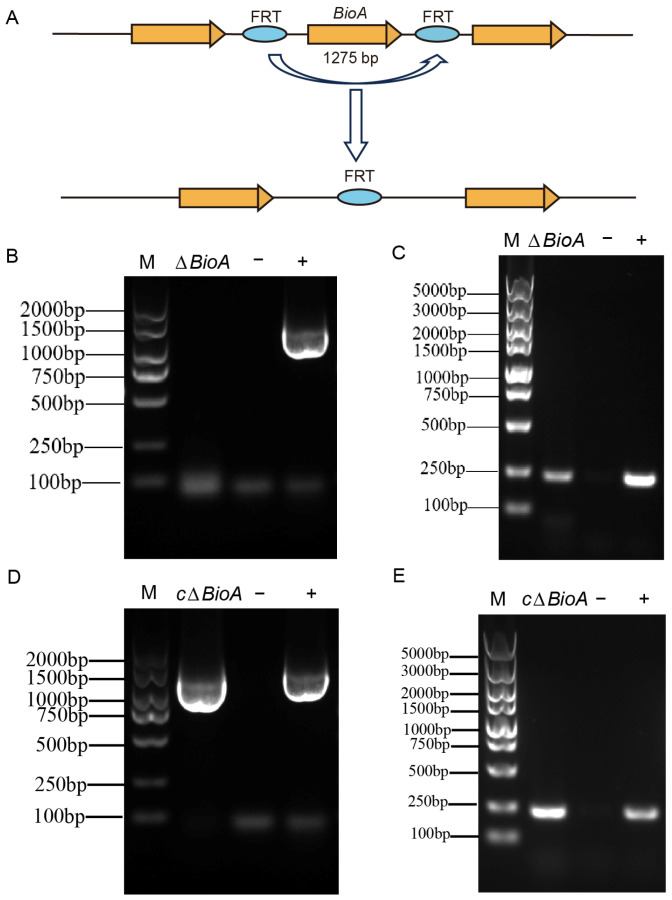
Construction and verification of a *∆BioA* mutant and complementary strain in *B*. *abortus* 104M. (**A**) Schematic diagram of the deletion of the *BioA* gene in *B. abortus* 104M. (**B**) The *B. abortus* 104M strain and the *∆BioA* mutant were identified by amplifying the *BioA* gene. (**C**) The species-specificity of the *B. abortus* 104M strain and the *∆BioA* mutant was determined by amplifying the *Bcsp31* gene. (**D**) The *B. abortus* 104M strain and the *c∆BioA* mutant were identified by amplifying the *BioA* gene. (**E**) The species-specificity of the *B. abortus* 104M strain and the *c∆BioA* mutant was determined by amplifying the *Bcsp31* gene.

To explore the role of biotin-synthesis-related genes in *Brucella* growth and infection, we constructed *ΔBioA* marker-free mutants using the homologous recombination method (Figure 2A); mutant strains lacking the remaining genes are currently under construction. For PCR identification, the *B. abortus* 104M-specific *Bcsp31* gene fragment and the fragments of *BioA* sequences with different sizes were successfully amplified, respectively, in the wild-type 104M strain and the *ΔBioA* mutant (Figure 2B,C), confirming successful deletion of *BioA* without residual resistance markers. The *BioA* gene was cloned into pBBR1MCS and electroporated into the *ΔBioA* mutant. PCR (Figure 2D,E) and sequencing confirmed the *ΔBioA*-complemented strain *cΔBioA* was successfully constructed.

### 3.3. Biotin-Free Environment and Deletion of the BioA Gene Limit the Growth of B. abortus 104M

**Figure 3:**
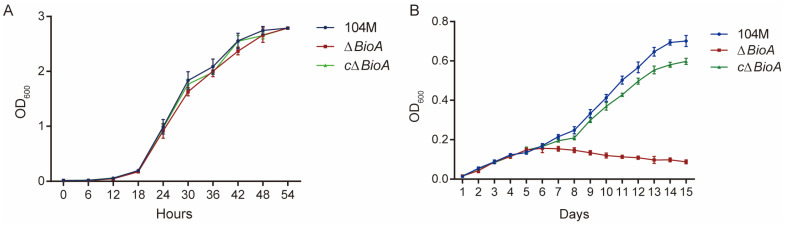
Growth curves of *B. abortus* 104M, *ΔBioA* mutant, and *cΔBioA*-complemented strain in TSB and biotin-free TSB media. (**A**) Growth curves in conventional TSB medium. (**B**) Growth curves in biotin-free TSB medium.

The *B. abortus* 104M, *ΔBioA* and *cΔBioA* were cultured in the normal TSB medium, and their growth curves were measured. It was found that they could grow in the medium, and there was no significant difference in growth (Figure 3A). The *B. abortus* 104M, *ΔBioA* and *cΔBioA* were cultured in the TSB medium without biotin. Compared with *B. abortus* 104M and *ΔBioA*, the *ΔBioA* mutant does not grow in a medium without biotin (Figure 3B), indicating that the absence of the *BioA* gene would severely affect the normal growth of the bacteria. Growth curve analysis revealed that the growth of *B. abortus* 104M and the *cΔBioA* mutant were severely impaired under biotin-free conditions (Figure 3B), suggesting that biotin serves as an essential growth factor for *Brucella*.

### 3.4. Observation Results of Electron Microscope

**Figure 4:**
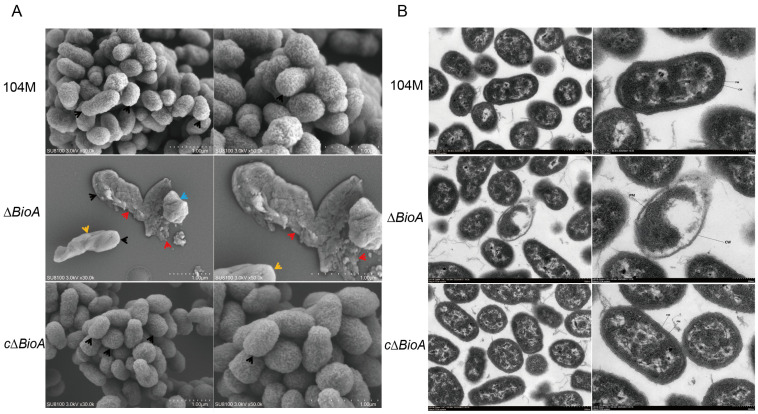
Morphological observation of *B. abortus* 104M, *ΔBioA* mutant, and *cΔBioA*-complemented strain by electron microscopy. (**A**) Morphology observed under scanning electron microscopy (SEM). (Key for arrow colors: black arrows indicate normal bacterial structures or elongated rod-shaped structures; orange arrows indicate bacterial surface collapse and wrinkling with intact cell walls forming small pits; blue arrows indicate rounded and significantly collapsed bacterial cells; red arrows indicate severely damaged, disintegrated bacterial cells with exposed matrix). (**B**) Morphology observed under scanning transmission electron microscopy (STEM).

The effects of *BioA* deficiency on the division, morphology, outer membrane structure and internal structure of *Brucella* were observed using scanning electron microscopy and transmission electron microscopy. Under a scanning electron microscope, the *B. abortus* 104M and *cΔBioA*-complemented strain exhibited uniform oval or short-rod shapes, intact cell walls, and smooth surfaces (indicated by the black arrow), presenting as oval or short rod shapes, with uniform size. The bacterial walls are intact, the surfaces are smooth, and there is no obvious wrinkling, collapse, or secretion structure. The number of the *∆BioA* mutant is relatively small, and the bacillus structures are more variable and elongated (indicated by black arrows), presenting a long rod shape. The overall damage is relatively obvious. Some areas are significantly collapsed and wrinkled (indicated by orange arrows), with the bacterial walls intact, forming small pits; a few have become round and are significantly collapsed (indicated by blue arrows), and in some severely damaged areas, a disintegrated state can be observed (indicated by red arrows) (Figure 4A). Under scanning transmission electron microscopy, the *B. abortus* 104M and *cΔBioA*-complemented strain are generally rod-shaped, with intact cell walls (CWs) that are uniform in thickness, and intact cell membranes (PMs) without any evidence of plasmolysis. The damage to the *∆BioA* mutant is quite obvious. Most of the structures are blurred, the intracellular protoplasm becomes pale, the shapes of the bacteria are irregular, some bacteria show obvious edema, the cell wall (CW) is locally dissolved and blurred, and the cell membrane (PM) is damaged (Figure 4B). In summary, compared with the wild strain, the outer membrane of the *ΔBioA* strain showed varying degrees of rupture, indicating that *BioA* deficiency would affect the outer membrane structure of *Brucella*.

### 3.5. Deletion of the BioA Gene Reduces the Intracellular Survival Activity of B. abortus 104M

**Figure 5:**
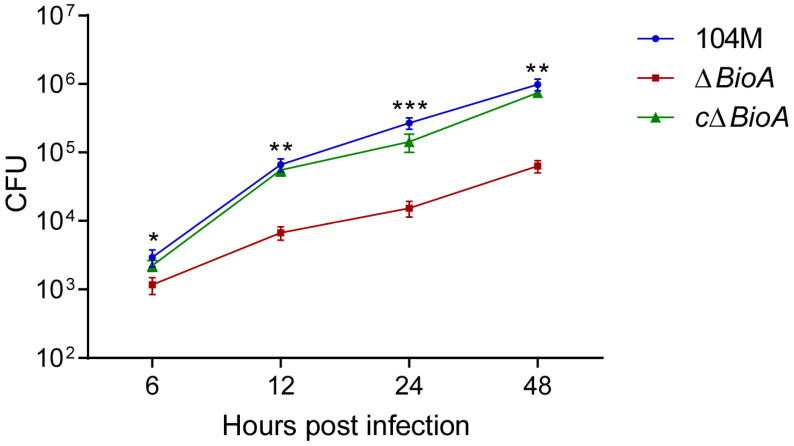
The intracellular survival of *B. abortus* 104M, *ΔBioA* mutant, and *cΔBioA*-complemented strain. Data are mean ± SD of triplicate wells (*n* = three independent experiments). Log10-transformed CFU were analyzed by two-way repeated-measures ANOVA followed by Sidak’s multiple comparisons test. * *p* < 0.05, ** *p* < 0.01, *** *p* < 0.001 vs. 104M at the corresponding time point.

We evaluated the impact of the *BioA* gene on the intracellular survival of *B. abortus* 104M in mouse macrophage RAW264.7 cells and found that the absence of the *BioA* gene led to a significant decrease in the survival ability of *Brucella* within cells. At 48 h post-infection, intracellular viable bacteria were quantified by plate colony counting. The survival rate of the *ΔBioA* mutant was only 5% that of the *B. abortus* 104M (*p* < 0.01; Figure 5). No significant difference was observed between the *B. abortus* 104M and *cΔBioA*-complemented strain. These results indicate that the *BioA* gene plays a critical role in the intracellular survival and replication of *Brucella* following host cell invasion.

### 3.6. The ΔBioA Mutant Strain Has Reduced Virulence in Mice

**Figure 6:**
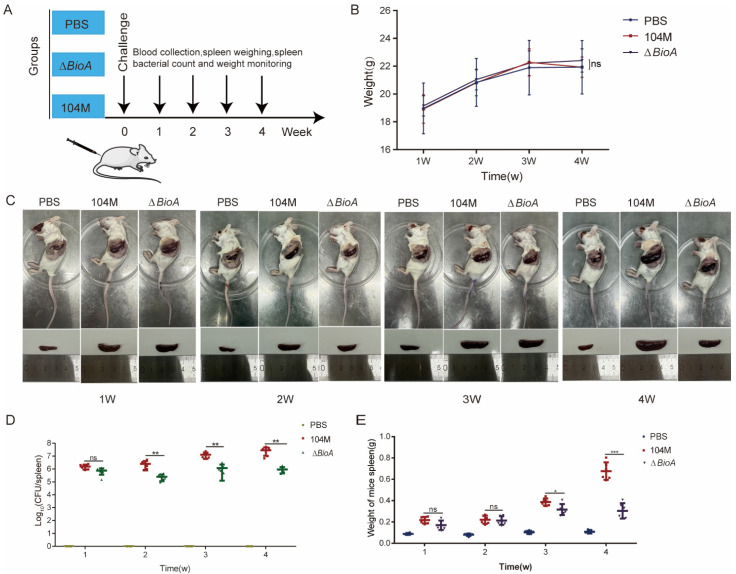
Mouse infection experiment. (**A**) Mouse infection experiment procedure. (**B**) The weight of the mice after 1 to 4 weeks of infection. (**C**) The spleens of mice infected for 1 to 4 weeks. (**D**) The bacterial load in the spleen of mice infected for 1 to 4 weeks. (**E**) The weight of the spleen of mice infected for 1 to 4 weeks. All data represent mean and standard deviation. * *p* < 0.05, ** *p* < 0.01, *** *p* < 0.001 were determined via two-way ANOVA followed by Sidak’s multiple comparisons test.

Mice were infected with the *B. abortus* 104M strain and the *∆BioA* mutant, and their median lethal doses were determined. The results showed that the median lethal dose of the *∆BioA* mutant increased to 5.0 × 10^9^ CFU, which was five times that of the *B. abortus* 104M strain (Table 1). To evaluate the role of the *BioA* gene in the process of infection with *Brucella*, we infected mice by subcutaneous injection, and systematically compared the colonization ability of wild-type strains with mutant strains in mice (Figure 6A). During the infection period, the body weights of the different groups of mice steadily increased, with no significant differences observed (Figure 6B). The dissection results showed that the spleens of mice in the *ΔBioA* infection group were significantly smaller than those in the *B. abortus* 104M group after the second week (Figure 6C). The viable bacterial load in the spleen was quantified by plate colony counting, and the results showed that the colonization ability of the *ΔBioA* mutant strain in the spleen was severely impaired. Compared to the wild-type infected group, the average bacterial load of the spleen of mice in the *Δ BioA*-infected group was significantly reduced in approximately the second week of infection (Figure 6D). At the same time, we measured the spleen weight of infected mice, and mice infected with the *ΔBioA* mutant strain showed significant spleen weight reduction, which is often associated with a reduced bacterial load, as well as a weakened inflammatory response (Figure 6E). These in vivo experimental results strongly suggest that the *BioA* gene plays an indispensable role in the immune clearance of *Brucella* against the host spleen.

### 3.7. The Content of Acetyl Coenzyme A and the Expression Levels of Virulence Factors in the ΔBioA Mutant Strain Decreased

**Figure 7:**
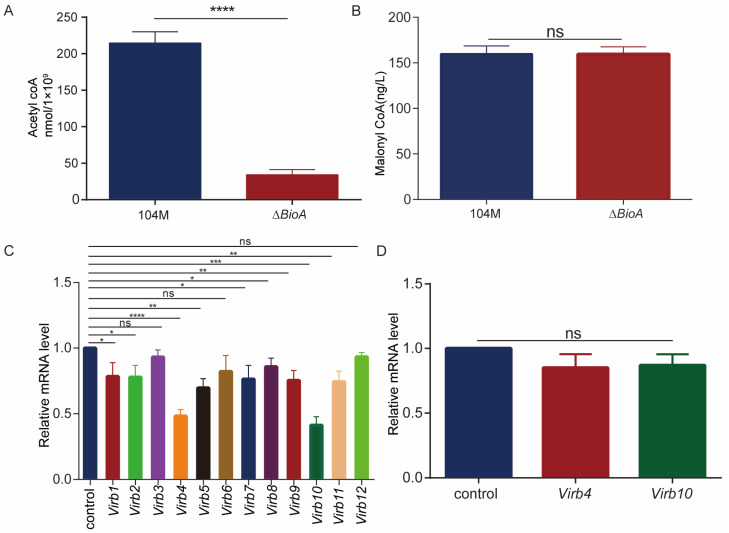
Detection of metabolites and virulence factors. (**A**) Determination of acetyl-CoA content in the *B. abortus* 104M strain and the *∆BioA* mutant. (**B**) Determination of malonyl-CoA content in the *B. abortus* 104M strain and the *∆BioA* mutant. (**C**) Expression levels of T4SS components in the *ΔBioA* mutant. (**D**) Expression levels of T4SS components in the *cΔBioA*-complemented strain. All data represent mean and standard deviation. * *p* < 0.05, ** *p* < 0.01, *** *p* < 0.001, **** *p* < 0.0001 were determined via one-way ANOVA followed by Dunnett’s multiple comparisons test.

Biotin indirectly affects the metabolic pathways of acetyl-CoA and malonyl-CoA, and the carbon skeletons of most lipid molecules (including the main components of cell membranes) are derived from acetyl-CoA and malonyl-CoA. We measured the content of acetyl coenzyme A in the *B. abortus* 104M and *ΔBioA* mutant strains. The results showed that the acetyl-CoA content in the mutant strain decreased significantly (Figure 7A). There was no significant difference in the content of malonyl-CoA between the *B. abortus* 104M and the *ΔBioA* mutant strain (Figure 7B). The *VirB1*–*12* in the type IV secretion system is an important virulence factor of *Brucella*. We compared the expression levels of virulence factors and found that the expression levels of virulence factors *VirB1*, *2*, *4*, *5*, *7*, *8*, *9*, *10*, and *VirB11* in the mutant strain were significantly reduced; in particular, the expression levels of *VirB4* and *VirB10* decreased the most (Figure 7C). The expression levels of the *VirB4* and *VirB10* of the *cΔBioA*-complemented strain showed no significant changes (Figure 7D).

## 4. Discussion

*Brucella* is a typical intracellular pathogen, and its pathogenicity depends on a sophisticated virulence regulatory network and adaptive metabolic plasticity within the host [23]. By constructing a *B*. *abortus* 104M *BioA* deletion mutant, this study provides the first direct evidence that the late-stage biotin synthesis pathway plays a central role in the core metabolic adaptation and pathogenic mechanism of *B*. *abortus*. To confirm the specificity of the mutant phenotype, we constructed a complementary strain (*cΔBioA*), which restored *BioA* function and related phenotypes to wild-type levels, thus confirming that the observed defects were specifically attributed to *BioA* deletion. Our results confirm that *BioA*, a key enzyme in biotin synthesis, serves as a critical metabolic hub linking biotin metabolism to bacterial pathogenicity by maintaining outer membrane structural integrity and regulating the transcription of genes encoding the type IV secretion system (T4SS). Loss of *BioA* function severely impairs the metabolic fitness of *Brucella*, leading to significantly reduced intracellular survival and in vivo colonization potential.

As a key catalytic enzyme in the late stage of biotin synthesis, the functional integrity of *BioA* directly determines the biotin synthetic capacity of *Brucella*. Deletion of *BioA* leads to growth arrest in biotin-deficient environments. Biotin is an essential coenzyme of acetyl-CoA carboxylase [24], and its deficiency may result in decreased acetyl-CoA levels. As a central precursor for fatty acid synthesis, energy metabolism and lipid biosynthesis, reduced acetyl-CoA directly limits the supply of phospholipids and lipopolysaccharides (LPSs) required for the bacterial outer membrane [25], which ultimately may lead to outer membrane structural damage. This finding provides metabolite evidence that biotin deficiency may mediate structural defects in the bacterial outer membrane. Thus, *BioA*-mediated biotin metabolism likely not only supports energy supply, but also regulates the biosynthesis and stability of the bacterial cell wall and outer membrane. Compromised structural integrity likely directly impairs the ability of the strain to resist host immune pressure [26]. In parallel, this study reveals a key regulatory role of *BioA* in T4SS-related gene expression: expression of VirB family genes, especially *VirB4* and *VirB10*, was significantly downregulated in the *ΔBioA* mutant. *VirB4* acts as the core ATPase of T4SS, providing energy for complex assembly and substrate secretion [27], while *VirB10* forms the transmembrane channel essential for outer membrane integration [27]. Downregulation of both genes suggests potential impacts on T4SS function. Combined with the phenotype of acetyl-CoA metabolic disorder, these findings suggest that *BioA*-mediated metabolic homeostasis may indirectly influence T4SS expression, potentially through alterations in central carbon metabolism and membrane integrity. *Brucella* may sense its nutritional status and environmental adaptability via the level of metabolites such as acetyl-CoA, and then regulate the expression of virulence genes to achieve a dynamic balance of metabolic adaptation and virulence activation. The nutrition-restricted environment of *Brucella*-containing vesicles (BCVs) in macrophages may further aggravate the metabolic defects of the *ΔBioA* strain, and the survival rate of the *ΔBioA* mutant in macrophages is significantly reduced (approximately 95% lower than the wild type). Consistent with previous research findings [22], the wild-type *B*. *abortus* 104M strain exhibited a typical median lethal dose (LD_50_), while the *ΔBioA* mutant strain had a significantly elevated LD_50_ of 5.0 × 10^9^ CFU. Relevant studies have confirmed that chronic colonization, rather than lethality, is the core biological characteristic of *Brucella* infection, so splenic colonization and spleen weight—which are more robust virulence indicators than LD_50_—were prioritized in this study to verify the virulence attenuation of the *ΔBioA* mutant strain [28]. These virulence defects are further reflected in the in vivo infection process, as the low biotin content in mammals [29] impairs the replication ability of the *ΔBioA* strain in the early stages of infection (such as in macrophages in local lymph nodes), reducing the number of bacteria that can spread to remote organs such as the spleen through decreases in blood circulation. After subsequent infection with spleen macrophages, the living environment of the *Brucella* contained in the vesicles will be worse, which will further inhibit the proliferation of residual bacteria, and ultimately lead to the bacterial load in its spleen being continuously lower than that of the wild-type strain.

Similar to the attenuation caused by *BioA* deletion in *Brucella*, the loss of biotin metabolic enzymes in *Elizabethkingia* and *Chryseobacterium* leads to biotin auxotrophy, significantly inhibiting biofilm formation and infectivity [25]. In *Mycobacterium tuberculosis*, mutants deficient in *BioA*, *BioF* or *BioB* display biotin auxotrophy and impaired intracellular survival and chronic infection [30]. *BioB* deficiency also blocks biotin synthesis in *Mycobacterium abscessus* and greatly reduces lung infection survival [31]. A *BioF* mutant in *Riemerella anatipestifer* exhibits biotin auxotrophy and abnormal cell morphology, resulting in reduced pathogenicity [32]. Notably, impaired biotin synthesis also weakens drug resistance and in vivo colonization in ESKAPE pathogens. Feng et al. demonstrated that deletion of the early biotin synthesis gene *BioC* in *Acinetobacter baumannii* and *Klebsiella pneumoniae* restores colistin sensitivity in *mcr-1*-harboring strains and reduces organ colonization in mice [33]. In *Pseudomonas aeruginosa*, *BioH* deficiency reduces carbapenem resistance and lung colonization, increasing mouse survival by 80% [29]. T4SS is the central virulence apparatus of *Brucella*, encoded by the VirB operon, and its function is essential for intracellular survival, replication and systemic infection [34]. Previous studies identified multiple regulators of T4SS, including *ArsR2*–*VjbR* [35], the *BvrR*/*BvrS* two-component system [36], and *QseC*/*QseB* and *Hfq* [27], which control VirB expression at the transcriptional level. This study identifies a novel, metabolism-dependent regulatory pathway mediated by *BioA*. The precise molecular mechanism linking *BioA* function to T4SS expression remains to be fully defined, but our findings expand and refine the T4SS-regulatory network and deepen the understanding of *Brucella* virulence control.

Based on the discovery of *BioA* as the core node in *Brucella* metabolism and virulence regulation revealed in this study, future research should focus on three aspects. Mechanistically, in-depth investigations are warranted to elucidate the specific molecular pathway underlying acetyl-CoA-mediated regulation of T4SS, should such regulatory crosstalk exist; mechanistic evidence should be established linking acetyl-CoA depletion to transcriptional modulation of the VirB operon; and alternative explanations including global stress responses, membrane instability, and compromised energy homeostasis should be experimentally excluded. Furthermore, systematic analysis of the interaction between *BioA* and the biotin-sensing system is required to delineate its functional role in chronic *Brucella* infection. In terms of application, we rely on the *ΔBioA* mutant to optimize attenuated vaccines, and at the same time develop small-molecule inhibitors targeting *BioA* to explore its synergistic effect with existing antibiotics. In addition, the functional conservation of *BioA* in different pathogenic species of *Brucella* should be verified, alongside improving the virulence regulation network of the biotin synthesis pathway, and providing theoretical and technical support for anti-brucellosis intervention.

## 5. Conclusions

Through multi-dimensional phenotypic verification and molecular mechanism analysis, this study clarified for the first time that *BioA* is a key connecting factor between biotin metabolism and the pathogenicity of *Brucella*. Its regulatory role may be achieved by maintaining the structural integrity of the outer membrane, and potentially indirectly regulating the transcription of T4SS-encoding genes. The realization not only deepens the understanding of the synergistic mechanism of intracellular pathogen metabolism virulence, but also identifies an anti-brucellosis intervention target that is both specific and necessary. As a unique biotin synthase of bacteria, *BioA* has low homology with host homologous genes, which gives it unique advantages in developing highly specific drugs or attenuated vaccines, providing a new direction for alleviating the drug resistance dilemma faced by brucellosis prevention and control.

## Figures and Tables

**Table 1:** The median lethal dose of the *B. abortus* 104M strain and the *∆BioA* mutant.

Bacteria	LD_50_
*B. abortus* 104M	1.0 × 10^9^
*B. abortus* 104M *ΔBioA*	5.0 × 10^9^

## Data Availability

For detailed data, please contact the author directly.

## Supplementary Materials

The following supporting information can be downloaded at https://www.mdpi.com/article/10.3390/pathogens15050473/s1, Table S1: Primers used in this study.
