# Supplementary material for: Exploring the Link Between Biotin Metabolism and Brucella Virulence: A Study on BioA
Source: Pathogens. 2026 Apr 27;15(5):473. doi: 10.3390/pathogens15050473 (PMC13209338; doi:10.3390/pathogens15050473)
Supplement: Supplementary file 1 [file pathogens-15-00473-s001.zip › Supplementary Table 1.pdf]

TABLE S1 Primers used in this study

Used for ΔBioA construction

Primers	Sequences (5'-3')	Usage
BioA-P1	ACCTGCAGGCATGCAAGCTT CGTTTGATCGTTACCGGCAC	To amplify BioA upstream fragment (104M)
BioA-P2	CGTGCCACTCCTGCGAAATCG	
BioA-P5	CGATTTCGCAGGAGTGGCACGCGGTCTGTTCC AGCAGGCTGGCCG	To amplify BioA downstream fragment (104M)
BioA-P6	ACCATGATTACGCCAAGCTT TTCGCTTGCATCGCGTTCAG	
BioA-F	ATGAACCCGAACACCTCGCC	Used for ΔBioA selection and verification
BioA-R	TCATCTTGTCTGCCCCGCAAT	
Bcsp31-F	TCGGTTGCCAATATCAATGCG	Used for ΔBioA selection and verification
Bcsp31-R	CGCTTGCCTTTCAGGTCTG	
BioA-Co-F	ttatcaggctctgggaggATGAACCCGAACACCTCGCC	Used for cΔBioA selection and verification
BioA-Co-R	CACTGATTAAGCATTGGTAATCATCTTGTCTGCC CCGCAA	

Used for qRT-PCR

Primers	Sequences (5'-3')
Putative X-F	CTTGCAGGCAGCTTATCTCA
Putative X-R	GCCGCATTCGTAAATCTGGA
BioB-F	GAAGATTGCGGTTATTGCAGC
BioB-R	CATTTGCAAGCGTATCGAGC
BioF-F	CGTGGTTGAAAGCATCTATTCC
BioF-R	TTGACGAGATAGTCGCACAG
BioD-F	CCGGAAGAGATGCATATTCC
BioD-R	TGTCTTCGTTCGCTTCACTG
BioA-F	CGCCGGATATTCTCTGTACAT
BioA-R	TTGAAAAAGGTGCGCAGCTTC
BioZ-F	TTTCCGCATCAGGCCAATATC
BioZ-R	TTCATACCTGCATCAGCACAG
16S-F	ACGTGCTACAATGGTGGTGA
16S-R	CAGAGTGCAATCCGAACTGA

Used for qRT-PCR

Primers	Sequences (5'-3')
VirB1-F	TGTTGCACCTCAGACTATGG
VirB1-R	CGTTTTTTTACTGGGGCAGC
VirB2-F	ATACGCACCCTCATTGTCTC
VirB2-R	GTATGTGCGTATAATTTCGGCG
VirB3-F	TGTTTGGGGTTCCTGTGATC
VirB3-R	GGTGCGTATGTGCGTATAAGG
VirB4-F	CCATCGCCTCAAACAATGTG
VirB4-R	ATTTGATTGCCGATTTCGTCCAG
VirB5-F	GATCTCTACGAAGCGGTGAT
VirB5-R	TTCGCGTCTAATTCGTGCTG
VirB6-F	TCGCAACTCGTCATAAGCTG
VirB6-R	GTAACAGTTCTGAAAAGCCCTG
VirB7-F	CTTCCTTGCCGGTTGCACTA
VirB7-R	GGTTTAGCCGGAACGCTT
VirB8-F	GTCCGGCTTGTCGAAAAATC
VirB9-F	CTTGCGTGCATACTGATCAC
VirB9-R	AATCGTACATGCGCTGATCTG
VirB10-F	ACCAAAGGACGATGTACTGG
VirB10-R	CGTCCACAGGACATAAATGC
VirB11-F	GGATCAGGATATTTCGGACAG
VirB11-R	TCAGCGCTTTCATCAGTGTG
VirB12-F	CCGCCCAAACAAACAGTCAA
VirB12-R	TTATAGATAGCCAGCGCACG
16S-F	ACGTGCTACAATGGTGGTGA
16S-R	CAGAGTGCAATCCGAACTGA
